# Commentary: Potential Links between Hepadnavirus and Bornavirus Sequences in the Host Genome and Cancer

**DOI:** 10.3389/fmicb.2018.01649

**Published:** 2018-07-23

**Authors:** Francesco Cetta, Maria Palmieri, Alessandra Renieri, Elisa Frullanti

**Affiliations:** ^1^IRCCS MultiMedica, Milan, Italy; ^2^Medical Genetics, University of Siena, Siena, Italy; ^3^Genetica Medica, Azienda Ospedaliera Universitaria Senese, Siena, Italy

**Keywords:** cancer susceptibility, lung cancer, oligogenic inheritance, whole exome sequencing, cancer genetics

In the paper by Honda et al. (Honda, 2017), sequences of non-retroviral RNA viruses seem to have been integrated into the host genome possibly by machineries of host retrotransposon, long interspersed nuclear element 1 (LINE-1, or L1). The integration events of viral sequences occur not only in somatic cells, but also in germ line cells and then can be inherited as EVEs (endogenous viral elements). Honda reports various possibilities for EVEs to have oncogenic potential and focuses both on HBV viruses for the occurrence of HCC and on endogenous bornavirus-like elements (EBLs).

Because EBLs are the only non-retroviral RNA virus-derived EVEs found in the human genome thus far, Honda focuses on the possible link between these elements and cancer, although the links have not been demonstrated.

Among the 7 Homo sapiens EBLNs (hsEBLN), hsEBLN-2 is most closely linked to cancer. Gene ontology analysis concerning *EBLN-2* suggests that it might be a tumor suppressor gene (TSG).

Interestingly, because mitochondrial dysfunction is found in cancer, hsHBLN-2 might play important roles in mitochondrial function and then act as a TSG. Honda correctly states that “Epidemiologic studies on the links between *EBL* mutations and cancers are clearly required. Furthermore, the causal relationship between such *EBL* mutations and cancers should be demonstrated in the future.” In particular, Honda quotes extensively one of our papers: “Whole exome sequencing (WES) using two sibling pairs of non-smokers with lung adenocarcinoma reveals that a truncated mutation in *hsEBLN-2* is only detected in affected siblings (Renieri et al., 2014). The authors concluded that this mutation in *hsEBLN-2* might predispose an individual to lung adenocarcinoma (ADCA)” (Honda, 2017). We indeed identified a frameshift mutation, the c.250dupA, p.N83fs in *EBLN2* gene (NM_018029) (Renieri et al., 2014). Interestingly, according to Honda, our report is the only study reporting a germline mutation of *hsEBLN-2* associated with tumor occurrence in the clinical setting.

We would like to make some comments, namely concerning our original approach to oligogenic signatures (i.e., the germline mutation of 5–8 genes) in the occurrence of common sporadic tumors.

We have used WES, associated with a discordant sibling strategy, to show that siblings sharing 50% of the genome, could differ for 5–8 germ line mutations, likely facilitating cancer occurrence in one sibling and its absence in the other. This is in line with the modern trend toward “personalized medicine,” i.e., each subject finds his/her personal way to cancer and every individual cancer is different from the other.

Therefore, we suggest caution before extrapolating that *EBNL-2*, a suspected tumor suppressor gene, could play a role such as APC germ line mutations in the occurrence of familial adenomatous polyposis (FAP) (Cetta et al., 2001)—or of other TSGs as causal responsible for inherited multitumoral syndromes—, in the occurrence of lung ADCA. EBLN-2 protein may be involved in regulation of tumor-related genes, in mitochondrial function and microtubule regulation (Figure 1A). Honda suggests that this mechanism could act as a pathogenic factor for the formation of a given tumor, namely lung adenocarcinoma, maybe according to “conventional” pathogenic pathways. In our study, on the contrary germ line mutation of the *EBLN* gene has been found as a part of an “oligogenic signature” responsible for tumor occurrence in a discordant sibling model, that aims at detecting the congenital basis for the emergence of a “personalized” tumoral variant, according to current knowledge concerning personalized medicine (Figure 1B).

**Figure 1:**
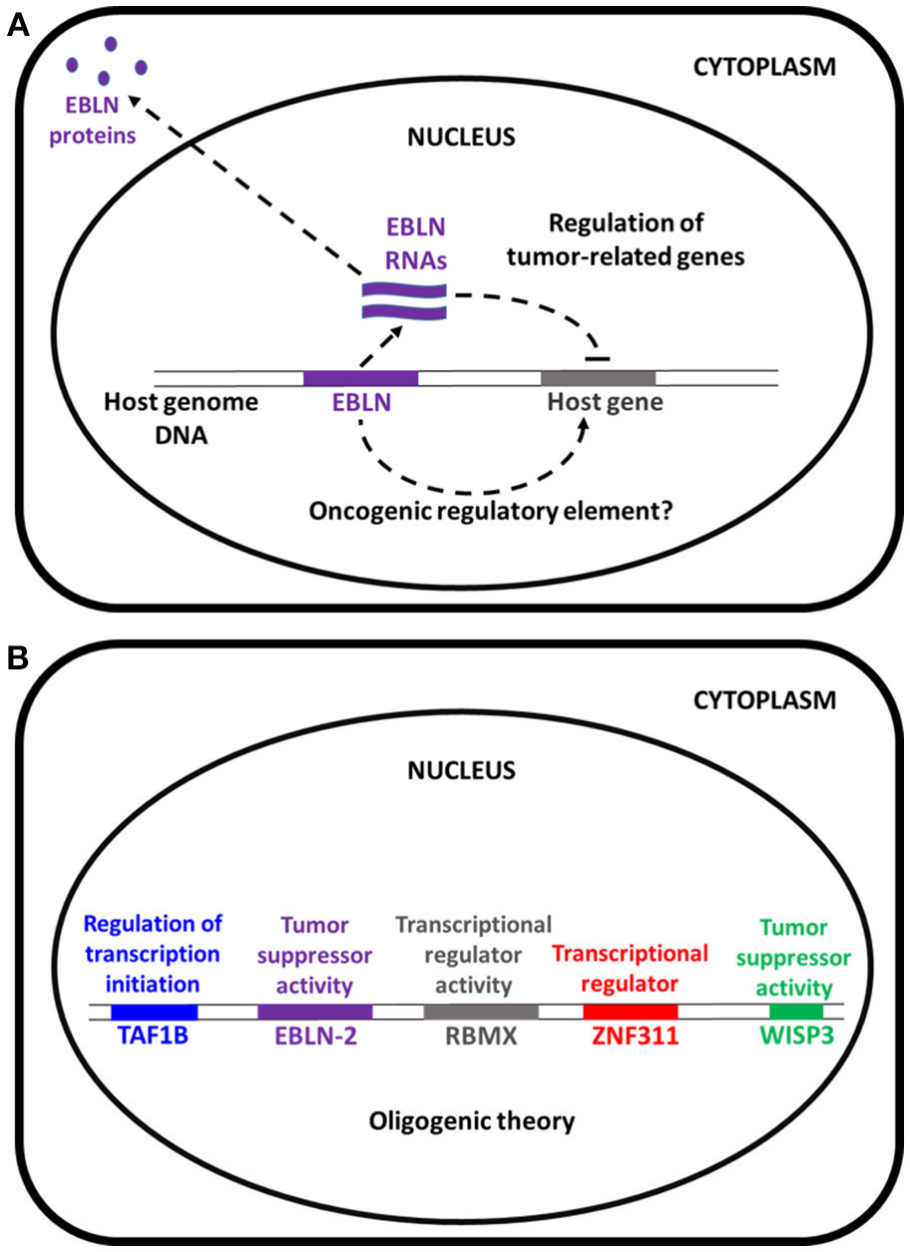
**(A)** Modified from Honda (2017). The hsEBLN-2 protein may be involved in mitochondrial function and might be a tumor suppressor. **(B)** EBLN-2 is involved in cancers together with others genes. For example in our study about lung adenocarcinoma susceptibility we found the involvement of TAF1b (component of RNA polymerase I core factor complex that acts as a GTF2B/TFIIB-like factor and plays a key role in multiple steps during transcription initiation), RBMX (RNA-binding protein that plays several roles in the regulation of pre- and post-transcriptional processes), ZNF311 (may be involved in transcriptional regulation), WISP3 (appears to be required for normal postnatal skeletal growth and cartilage homeostasis, and WISP3 regulates accumulation of cellular reactive oxygen species) as a proof-of-concept of the oligogenic theory (Renieri et al., 2014).

Our study is based on a philosophy that is completely different from the “gene hunting” approach of 30 years ago, aimed at detecting a single gene responsible for a single cancer. Namely, we have already found a peculiar oligogenic signature, responsible for lung ADCA in six additional discordant siblings (Cetta et al., 2017). Interestingly, a total of more than 50 different genes have been detected as “facilitating germ line mutations” in oncogenes or TSG in the entire series of eight patients with lung ADCA. They are responsible for the formation of a “personal” cancer, even if they could not include the single main determinant of tumor occurrence. Similar findings have also been detected in a non-smoker with lung squamous cell carcinoma (Baldassarri et al., 2018).

We hope that this comment could help to clarify better the significance of our findings. EBNL2 is not a gene specifically causing the formation of lung adenocarcinoma, but a germline mutation that may facilitate its occurrence as a component of a tumor predisposing oligogenic signature, at individual level. While waiting for more stringent causal relationships between EBLN-2 and various types of cancers, we think that the present clarification could prevent misleading interpretations.

## Author contributions

FC, MP, and EF wrote the manuscript. MP performed Figure 1. EF and AR made substantial contributions to the manuscript idea. All authors have given final approval of the version to be published and agree to be accountable for all aspects of the work.

### Conflict of interest statement

The authors declare that the research was conducted in the absence of any commercial or financial relationships that could be construed as a potential conflict of interest.

## References

[B1] Baldassarri M. Fallerini C. Cetta F. Ghisalberti M. Bellan C. Furini S.. (2018). Omic approach in non-smoker female with lung squamous cell carcinoma pinpoints to germline susceptibility and personalized medicine. Cancer Res. Treat 50, 356–365. 10.4143/crt.2017 28546520 PMC5912139

[B2] Cetta F. Montalto G. Gori M. Curia M. C. Cama A. Olschwang S. (2001). Germline mutations of the APC gene in patients with familial adenomatous polyposis-associated thyroid carcinoma: results from a European cooperative study. J. Clin. Endocrinol. Metab. 85, 286–292. 10.1210/jcem.85.1.6254 10634400

[B3] Cetta F. Renieri A. Frullanti E. (2017). Germline mutations in lung cancer and personalized medicine. Fam. Cancer 17, 429–430. 10.1007/s10689-017-0044-4 28914390

[B4] Honda T. (2017). Potential links between hepadnavirus and bornavirus sequences in the host genome and cancer. Front. Microbiol 19:2537 10.3389/fmicb.2017.02537 PMC5742130 29312227

[B5] Renieri A. Mencarelli M. A. Cetta F. Baldassarri M. Mari F. Furini S.. (2014). Oligogenic germline mutations identified in early non smokers lung adenocarcinoma patients. Lung Cancer 85, 168–174. 10.1016/j.lungcan.2014.05.020 24954872

